# The integration of blended learning into an occupational therapy curriculum: a qualitative reflection

**DOI:** 10.1186/s12909-017-0977-1

**Published:** 2017-08-17

**Authors:** Paula Barnard-Ashton, Alan Rothberg, Patricia McInerney

**Affiliations:** 1 School of Therapeutic Sciences, Faculty of Health Sciences, University of the Witwatersrand. Affiliated to the Department of Occupational Therapy, 7 York Road, Parktown, Johannesburg, 2193 South Africa; 2 School of Therapeutic Sciences, Faculty of Health Sciences, University of the Witwatersrand, 7 York Road, Parktown, Johannesburg, 2193 South Africa; 3 Centre for Health Science Education, Faculty of Health Sciences, University of the Witwatersrand, 7 York Road, Parktown, Johannesburg, 2193 South Africa

**Keywords:** Blended learning, Curriculum development, E-learning, Occupational therapy, Reflective practice

## Abstract

**Background:**

This paper presents a critical reflection of the integration of Blended Learning (BL) into an undergraduate occupational therapy curriculum which was delivered through Problem Based Learning (PBL).

**Method:**

This is a qualitative reflection of a Participatory Action Research (PAR) study using Brookfield’s model for critical reflection of an educator’s practice. The model uses four ‘lenses’ through which to focus enquiry: Lens 1) our autobiography as a learner of practice; Lens 2) our learners’ eyes; Lens 3) our colleagues’ experiences; and Lens 4) the theoretical literature. Grounded theory analysis was applied to the data.

**Results:**

The factors that contributed to successful integration of technology and e-Learning into an existing curriculum, the hurdles that were navigated along the way, and how these influenced decisions and innovation are explored. The core categories identified in the data were “drivers of change” and “outcomes of BL integration”. Key situations and pivotal events are highlighted for their role in the process that led to the project maturing. Each lens reflects the successes and hurdles experienced during the study.

**Conclusion:**

Brookfield’s model provides an objective method of reflection which showed that despite the hurdles, e-Learning was successfully integrated into the curriculum.

## Background

Health science educators in South Africa have been under increasing pressure to produce greater numbers and higher quality health care practitioners within the massification trend [1]. Statistics show that fewer than 50% of students progress through their degree courses in the minimum number of years. The majority require an additional year or two, drop out or are excluded before completion [2]. As class sizes increase, a greater burden is placed on the higher education system [1]. Clinical fieldwork placements must also accommodate more students [3]. Meanwhile, lecturer numbers have not increased, resulting in higher workloads to support the undergraduate curriculum. Some curricula are very demanding in terms of student:lecturer ratios. The consequence of lecturers having to focus on undergraduate teaching and supervision is the erosion of time for high quality research.

This study focuses on an occupational therapy curriculum, which is a 4 year Bachelor of Science degree delivered primarily through Problem Based Learning (PBL) [4, 5]. The average PBL session involves a lecturer:student ratio of approximately 1:9. Producing skilled practitioners requires clinical skills training and individual supervision at dispersed clinical fieldwork sites. Faculty thus have to travel to various clinical fieldwork sites to support this intensive training process [3, 4]. Over the last decade the full time staff establishment has remained static at eleven qualified academics.

In 2008, the Department of Occupational Therapy (DOT) held a strategic planning meeting at which the teaching and learning strategy was reviewed. Brainstorming on how to cope with the rising student numbers and alignment with the institution’s plans led to the agreement that e-Learning in a Blended Learning (BL) design was needed. This set the stage for the development of a BL project to drive the integration of e-Learning into the undergraduate curriculum.

The process of initiating the e-Learning project was enabled by the successful application for grant funding to support staff and infrastructure development. At the time, the university was investigating moving away from a proprietary Virtual Learning Environment (VLE) to an open source VLE, but in the absence of any firm decision, an interim open source VLE was set up on a local Faculty server. This was branded “e-OT”, which soon became embedded in the vernacular of the students and lecturers of the DOT. Students and lecturers were trained on the use of the VLE and e-OT was launched at the start of the 2010 academic year.

### Birds eye view of the integration of BL into the OT curriculum

The constructivist nature of PBL in medical education creates an appropriate foundation to be extended by the introduction of BL to the course delivery [6, 7]. One of the most commonly cited early definitions of BL is by Graham [8], “Blended learning systems combine face-to-face instruction with computer mediated instruction” (page 5), which he argues is encompassing of a variety of categories and approaches to BL but not that general as to apply to all learning modalities. This definition has expanded over time to acknowledge the integration of pedagogical approaches, a shift towards student-directed learning, student peer-to-peer collaboration, and accommodation of different student learning styles [6, 9, 10] which are all common traits with PBL [7].

A medium impact approach (components of the course are selected for development as online activities based on pedagogic merit) [11] to a program level [8] integration of BL was used over a 6 year Participatory Action Research (PAR) study. Savin-Baden and Wimpenny [12] describe PAR as having the goal of “creating knowledge to inform action” and is “conducted *with* people as opposed to *on* people”. PAR is an iterative process of planning, acting, measuring, and reflecting/reviewing to influence the follow-up cycle [12, 13], with reflexivity that emerges from continuous in-action reflection [14].

Lecturers of the DOT are typically responsible for managing three to five problem scenarios within their field of interest, as well as the associated skills laboratories and clinical fieldwork, across the program. The problem scenarios were all developed and well established prior to this study [3–5]. During 2009, the lecturers and the Project Manager (PM) held curriculum development workshops (Table 1) and the PM attended curriculum meetings throughout the project to provide feedback and design new intervention strategies in line with the PAR cycles.

**Table 1:** The introduction of BL over 6 years

2009	• Established the e-Learning team (the PM and a graphic designer). • Lecturer workshops on BL, the VLE tools and curriculum integration strategies.
2010	• Established a mini in-house computer centre with 10 computers and PBL rooms each had a computer and data projector installed. • The VLE was branded “e-OT”. • Curriculum guideline books were issued on an interactive HTML based Compact Disk (CD) (previously a CD of Rich Text files). • All student groups attended computer laboratory based training on the VLE and online resources. • Online class tests were designed and administered for two occupational science modules. • Wiki’s were created on the VLE for PBL group collaboration. • The 3rd year paediatric learning difficulties problem scenario was redesigned to include group wiki’s, a formative assessment quiz, and interactive lessons. • Developed an eBook for the 3rd year psychosocial module.
2011	• TCD’s issued to students for 4th year rural fieldwork with pre-paid data to facilitate access to the VLE. • Introduction of responders and an interactive whiteboard in the main lecturer theatre. 10 additional computers were installed in the in-house library. • Lecturer shared cloud based folder for department documents and editing curriculum documents. • Increased use of formative quiz, online class tests and written assignments submitted via the VLE across all levels of the curriculum. • The e-Learning team appointed an Information Technology (IT) technician.
2012	• The server hosting the VLE was upgraded to cope with the increased traffic. • Lecturers issued TCD’s: mark student fieldwork, improve communication, present face-to-face content. • Introduction of submitting video assignments for 1st year interview roleplay. • The e-Learning team appointed an instructional designer (who was also an occupational therapist and lecturer).
2013	• The curriculum books were migrated from the interactive CD’s onto the VLE. • Clinicians and external fieldwork supervisors were given access to the VLE areas applicable to their engagement with the students. • Majority of written assignments were submitted via the VLE and plagiarism scanning software. • International experts were live video streamed for upskilling workshops with the lecturers and postgraduate students on topics such as Evidence Based Practice and using Rache analysis in research.
2014	• Increased development of interactive multi-media lessons on the VLE for PBL modules in 2nd, 3rd and 4th year. • Introduction of gamification in the “Who wants to be an ethics millionaire” game on the 4th year VLE. • Students started using the data projector in each PBL session to collaborate electronically during the PBL process (replaced scribing on poster paper).
2015	• WebQuest to orientate 1st year students to the VLE and university online resources. • Lecturers created podcasts to cover difficult content in 3rd and 4th year curriculum. • Piloted the use of live video calling for supervision and case presentations for students at remote fieldwork sites. • Students shared online resources via discussion forums and wiki’s.

Lecturers were encouraged to use the time they typically use in preparing for their PBL scenarios to consider content that could be learnt through the online activities, and consider the pedagogical value of the activity over prior content delivery methods. Student learning hours allocated to the PBL scenario remained unchanged; with the problem scenario introduction sessions, mid-problem tutorials and problem feedback sessions remaining face-to-face and students having sufficient resource time to complete the online activities and address the problem learning objectives. Table 1 provides an overview to the integration of BL into the PBL curriculum over the 6 years of the project. These activities were reviewed, updated and rolled-over to each consecutive year. Students had access to the VLE of their prior academic years as they progressed.

## Method of reflection

Critical reflection is the process of interrogating our experiences, questioning our assumptions, illuminating sociocultural power-relationships, postulating change and creating new action [15–17]. In health education, there is large emphasis on developing critical reflection skills in our students through the introduction of reflective journals and learning portfolios [15, 18, 19], particularly within PBL [20], but less focus is placed on health educators being critically reflective of their own teaching and learning practice. Mann, Gordon and MacLeod [19] in their systematic review of reflection and reflective practice in health education failed to even consider the value of the reflective practice of the health educator. Brookfield [16, 21] presents four lenses through which critically reflective practice in teaching can be investigated. Oldland [22] used this model to reflect on her professional development as a nurse educator in a structured format. The Brookfield model [16, 21] resonated with the scope of this study as it represented the perspective of each group of stakeholders; namely the autobiographical lens of the primary investigator, the lens of the students and the lens of the lecturers. The fourth lens considers the research literature and theory underpinning the study, in this context it pertains to blended learning and connectivism. Table 2 summarises the evidence contributing to each lens.

**Table 2:** Evidence considered in each of the Brookfield lenses

Brookfield (1998) Lens:	Evidence:
Lens 1: autobiography as a learner	Occupational Therapy BSc (1994) and MSc (2009): PM degrees attained through the DOT Online notebook collection of experiences and thoughts during the course of the study: Tweets of content; pictures of interesting observations; field notes Audio records of meetings, congress sessions, paper presentations, courses and workshops Prior learning – mother Computer Science teacher, learnt web design principles when managing an online publication Roles: Faculty Information and Communication Technology Committee chair; VLE audit workgroup member Awards: Vice Chancellor’s team teaching award (2010); South African Association of Health Educators (SAHEE) congress poster prize (2012)
Lens 2: our students’ eyes	Survey data: 3rd and 4th year students’ experience of e-learning VLE Footprint: access and uptake of using the VLE Anecdotal commentary: students emails / conversations with the e-Learning team
Lens 3: our colleagues’ experiences	Minutes of meetings: curriculum review and strategic planning meetings of the DOT Survey data: Lecturer use of iPads (2012 and 2013) Reflective interviews and focus groups: Individual interviews (2012), focus groups (2015) Performance effect: Paediatrics module and Psychosocial module analysis of student marks and pass rates VLE Footprint: access and uptake of use of the VLE by lecturers over time Anecdotal commentary: emails, corridor conversations
Lens 4: theoretical literature	Books and journal articles within the following topics: • Connectivism • Participatory Action Research • Blended learning • E-Leaning • Mobile learning and mobile technologies • Education theory • Problem Based Learning

### Stakeholders

The Project Manager (PM) is the primary investigator in the study (Lens 1). She is an occupational therapist with a master’s degree, pursuing a doctorate through the current study. She was a member of the DOT lecturing staff for 6 years prior to taking the position as the e-Learning PM. The e-Learning team started with the PM and a graphic/web designer, and expanded during the study to include an IT technician and instructional designer. The instructional designer was also an occupational therapist with a master’s degree, and lecturer with an interest in BL.

The students (Lens 2) in the study were all undergraduate occupational therapy students from 2010 to 2015 (Table 3) who consented to participate in the research activities indicated in Table 1. The PM was not involved in any teaching or assessments of the undergraduate students of the DOT apart from introducing the students to the VLE and supporting their BL needs. She thus had no direct influence on student performance. Over time most students didn’t even realise that the PM was a previous lecturer of the DOT.

**Table 3:** Number of students first accessing and repeating each course level per academic year

	First year First access (Repeat)	Second year First access (Repeat)	Third year First access (Repeat)	Fourth year First access (Repeat)
2010	61 (0)	37 (1)	29 (3)	37 (2)
2011	37 (2)	40 (1)	41 (2)	26 (9)
2012	54 (0)	44 (0)	36 (6)	33 (0)
2013	45 (2)	41 (1)	40 (5)	38 (0)
2014	62 (0)	47 (0)	33 (8)	37 (0)
2015	61 (2)	48 (1)	39 (4)	34 (1)

There are two groups of lecturer participants in this study (Lens 3). The first group includes the 11 lecturers who were part of the inception of the BL project prior to the start of the 2010 academic year. This group will be referred to as lecturers. The second group includes five lecturers who joined the DOT through natural staffing turnover after the BL project was in process. This group will be referred to as the “ensuant lecturers”. The PM conducted all the interviews and focus groups with the lecturers (Lens 3).

All stakeholders are considered possible participants in the study as is typical in PAR, thus no sampling strategy was applied. All lecturer stakeholders were active researchers within the PAR study contributing to all phases of the PAR cycles [13]. All lecturers and students were invited to participate in the various data collection activities described in Table 2. Those who consented to participate were included in the data collection activity. All participants were informed that there was no prejudice or risk to non-participation at any point and any-time withdrawal of consent to use data records was given.

### Data collection

Nine individual lecturer interviews were conducted during two to three-hour prearranged appointments in the DOT at the start of 2012. These were the lecturers who were part of the inception of the project and still members of the DOT after 2 years. The interviews were open ended and probing in nature, allowing the participant freedom to reflect on the development of BL within the occupational therapy curriculum over the prior 2 years.

All lecturers were invited to attend a two-hour focus group which took place in a meeting room at the DOT in mid-2015. They consisted of a group of four lecturers and a group of four ensuant lecturers. The focus groups were open-ended discussions of their experiences of BL.

Students were asked to complete electronic surveys at the end of the computer laboratory sessions dedicated to introducing and updating students to the VLE and BL resources. Informed consent was given by participants and students were under no obligation to participate. Participation rates were consistently over 65%. Responses to the short open questions were included as data for this paper.

Data such as the anecdotal commentary, meeting minutes, awards, experiences and personal reflections of the PM were electronically captured in an online journal.

### Data analysis

The interviews and focus groups were audio recorded and orthographic transcriptions [23] were produced by an external transcriber who has experience in transcribing medical and health related data. Transcriptions are not publicly available due to the small confined stakeholder group where full transcripts may have multiple identifiers placing confidentiality at risk. Strauss’s grounded theory method [24] was used to code and analyse the collected data. The transcriptions and journal data were open coded using MaxQDA version 11 initially and migrated to version 12 [25] by the PM. Axial coding and constant comparison were applied to integrate the data and form the theory [14, 23, 24]. The coded files were reviewed by the e-Learning team’s instructional designer as she has similar qualifications and experiences to the PM. This review was a random check of the coding consistency across the files, followed by a discussion and agreement on the code patterns and themes.

## Results

Through Strauss’s grounded theory analysis [24] of the data presented in Table 2, two core categories were interpreted: 1) Drivers of change and 2) Outcomes of BL integration. The results present the evidence supporting the two core categories from the perspective of each of the Brookfield [16] critically reflective lenses, in order to give voice to each of the stakeholder groups within this PAR study.

### Critically reflective lens 1: Autobiography as a learner (*project manager’s lens*)

Reflecting on professional experiences is subjective and hinges on the psychosocial state of the individual at the time. An autobiography is typically a recounting of life events that has brought an individual to a particular level of understanding and belief structure surrounding the topic of concern. Brookfield [16] specifically suggests that educators reflect on their own learning experiences, which have informed the way they craft their education practice. The experiences presented here have shaped the decision making and change facilitation process during the study.

#### Buy-in and goodness of fit (Core category 1: Drivers of change)

The Project Manager (PM) had studied occupational therapy as a student in the DOT at both undergraduate and postgraduate levels, and subsequently joined the DOT lecturing staff. As an undergraduate student, the curriculum was delivered in a traditional lecture based format. The DOT transitioned to PBL before the researcher entered the DOT as a lecturer. The PM observed that PBL required both independent and group based learning allowing for the integration of BL without disrupting the timetable or basic structure of the curriculum. Institutional knowledge and a good understanding of the occupational therapy curriculum as a former student and current lecturer, enabled the PM to gain the trust of lecturers and students in the DOT. This trust opened doors to comprehensive buy-in to the BL concept and willingness to adapt the existing curriculum.

Nurtured by a mother who is a primary school teacher contributed to an innate foundation of teaching and learning practice for the researcher.

Journal entry (2013): “I am not sure that I would have been able to do this job if mom hadn’t chosen to get her teaching certification in computer literacy. I have a big advantage in growing up with home based computing skills ahead of my peers.”

While in private practice, the PM developed a remedial and special education website and thus gained exposure to web design and online tools. This was largely self-taught, leading to confidence in being able to develop basic e-Learning skills, which could then be transferred to the lecturers of the DOT.

Lecturer A, anecdotal commentary (2010): “We trust you – you know far more about this stuff than we do”

The lack of formal training in education theory and practice was however a concern for the PM, creating some anxiety at being responsible for change management that would have to be evidence based. The PM entered a fellowship programme in health science education (Sub-Saharan Africa - FAIMER Regional Institute [26]), which focused on building a community of health science educators, producing education research and developing the pedagogical skills of the fellows.

The researcher was consensually considered the “best man for the job” from the start of the project, illustrating a general goodness of fit, which resulted in strong buy-in from the lecturers of the DOT.

#### Just do it (Core category 1: Drivers of change)

Decision making is often time sensitive. At the inception of the project it became apparent that the institution was changing the VLE and there was uncertainty about the timelines of the rollout of this change. Lecturers can be change averse, especially when it comes to understanding technology and software. Concerns were raised about having to start on one system and then migrating to an unfamiliar system. It was agreed that waiting would be a risk, which drove collaboration with the Faculty IT unit to house an interim VLE on a local server. Five years and two institutional VLE’s later, the project is still on the same Faculty hosted VLE (though upgraded both in terms of server and software updates). The project started because a plan was made to “just do it”.

Resilience was a trait used to describe the PM’s approach to driving the process of integrating BL and maintaining momentum, creating a general ethos of “just do it”.

Lecturer B, anecdotal discussion (2015) about the PM: “You are tenacious and energetic, as in excited about the project and able to enthuse even the most obstinate naysayer. And it meant that despite some academic staff who were reluctant to try new things and scared of all things technology, you were able to get everyone on board and engaged in the project to the extent that these very hesitant/reluctant people are now running the content mostly by themselves. So, you have thus been able to ensure the sustainability of the program.”

#### Support (Core category 1: Drivers of change)

Management support lent credibility to the project for all stakeholders and mentored the PM in the prioritisation of decisions and management tasks at every point during the project. Financial constraints are regularly cited as a limitation to the success of a project [27]. This project was awarded substantial grant funding due to support by Faculty management. The budget has increased over time due to the perceived success of the project, allowing for expansion of the e-Learning team, facility upgrades, Wi-Fi coverage of the building housing the DOT, increase in the number of computing terminals for student use, and provision of Tablet Computing Devices (TCD’s) for lecturers. The funding allocated to this project afforded the technology enhancements that optimised the quality of BL integration.

The launch of the VLE was on a test server with relatively low processing power as the IT support services were cautious about the rollout of e-Learning. The system was initially stable, but as adoption of BL increased and lecturers became willing to try new methods of teaching and assessment, server capacity became an issue. This culminated in a system ‘crash’ during an online assessment when simultaneous student access overloaded the server.

Lecturer B, personal correspondence (November 2010): “Backend glitches make the lecturer look incompetent in front of students. This produces a reluctant technology adopter. A very big barrier.”

The VLE has since migrated to larger servers, twice, in response to the increased uptake of BL and the variety of plugins and online tools being actively integrated. Although there were initial challenges of server space, the Faculty IT unit has provided ongoing back-end support to the project. This highlights the risk factor if IT support is inadequate and how in this project has assisted with the sustainability as the PM has no server coding, setup or maintenance skills.

#### Valued as a game changing project (Core category 2: outcomes BL integration)

The success of a project can be evaluated by its recognition as a ‘game changer’ or when it stimulates groups outside of the target population to seek support for adopting the model set by the project. In October 2010, the project was awarded the Vice-Chancellor’s team teaching award, a mere 10 months after inception. Early recognition at that level created a sense of belief in the project by all stakeholders and a determination that any hurdles could be taken in stride.

The PM was selected to be a member of the University VLE audit workgroup, further demonstrating that the project was respected for its advances and that the PM could contribute to the University’s decision making process.

Journal entry (2012): “A pivotal moment for me was at a Faculty function where [the Dean of the Faculty] pulled me aside to tell me that she saw me as a younger version of herself and that she was proud of the impact that I have made within the faculty. This brief moment in time has fuelled my confidence in what I am doing”.

These experiences and learning opportunities have shaped the PM’s lens. However, technology advances rapidly, which creates new challenges and opportunities for adaptation and innovation in BL. The prominent skill learnt is the ability of the PM to evaluate advances for value to the project, or to confidently reject those deemed less relevant.

### Critically reflective lens 2: our learners’ eyes (*the students’ lens*)

The students are the end-users of BL and the focus should therefore be on improving their learning experiences to enhance their academic performance. Critical reflection of their experiences can only be deemed true through their ability to give feedback anonymously [16]. This translates into responsive adaptation of BL according to the needs and preferences of the students. All surveys were anonymous, with the only identifier being the year of study.

#### Primed for blended learning (Core category 1: Drivers of change)

There is consistent evidence that the students enter the degree with the computing and online skills necessary to cope with BL [1]. Despite the early concerns of lecturers that students would be ill prepared for the digital literacy demands of BL, a survey of the third-year occupational therapy students during 2011 (*N* = 43), the second year of the study, indicated that 91.2% of the 34 respondents had access to computers during high school and 88.2% reported having a personal use laptop by the time of the survey.

In one class a student created a personal web page for the class to share information so that they could have a private work-ground.

Ensuant lecturer A, focus group (2015): “She has made her own page as the class rep and any documents that I put up on e-OT she will also put up on the page. So I think she is a bit of techie whizz but she has […], her own personal website. And because I don't have access to said page which is fine, and I shouldn't have, […] and they can be completely honest with each other whereas obviously on e-OT there is a degree of caution and censorship you know; […] So they have really taken this whole e-Learning thing on board.”

Lecturer C, personal discussion (2013): “I often have students say to me ‘Just put it on e-OT, ma’am’.”

Both the quantitative and qualitative data reflect that the students are entering the curriculum primed for BL, and they are starting to adapt and innovate towards their own preferred BL tools.

#### Feeling reassured (Core category 2: outcomes BL integration)

The emergence of the feeling reassured theme through the lens of the learner showed two distinct sub-themes. For themselves, it was viewed as an enabler and a positive factor. Counter to that was a perception that for the lecturers the VLE provided a fall-back, and was thus a negative factor in the eyes of the students.

Having a VLE seems to provide the students with a feeling of reassurance in that all their curriculum information is in one ‘go-to’ place. It is accessible around the clock and maintains the history of the year-to-date. Three primary indicators were: a) students expressed that they access e-OT when they are unsure of something; b) access first thing in the morning to see if there is anything new that they should know for the day; and c) the access footprint indicated that students consistently access more frequently in the month prior to examinations suggesting that it forms part of their study pattern. The following quotes from students reflect their relative dependence on e-OT and how it is perceived as making them feel more secure in their day-to-day curricular activities.

4^th^ year student A, anecdotal email (2014): “We rely on e-OT, especially before exams.”

3^rd^ year student A, survey (2010): “I like that it's easier to access than notice boards and I can check things whenever I am unsure or have time.”

Students also perceive that some lecturers use the VLE as a fall-back for when contact time is not enough to complete a lecture or contact is cut it short in order to do something regarded as more important. Students’ complaints about this were regular in the initial phases of implementing BL, as reflected by the following two student comments towards the end of the second year of e-OT.

4^th^ year student B, survey (2012): “It is a dumping ground for unfinished business”

3^rd^ year student B, survey (2010): “The lecturers tend to abuse it in that they use it as an excuse for rushing through a lecture. YES this REALLY bothers me! ‘Specially coz I do listen in class… mostly.”

As the lecturers have become more proficient in the integration of BL this issue seems to have diminished but is still worth noting for the early stages of a similar project. The implementation of BL in the curriculum seems to have given the students a sense of reassurance both in terms of freedom to express their opinions and in knowing that their learning materials are always accessible.

#### Communication (Core category 2: outcomes BL integration)

There is a strong sense that having a VLE has facilitated both peer-to-peer and student-lecturer communication. Prior to and in the early stages of e-OT the student classes created a shared email account to which they emailed course notes, notices and other information. Each student then accessed the class email (via common username and password) in order to get the information. It was their self-created online information sharing space. For more urgent notices a mobile phone instant message tree was created so that lecturers could message class representatives who would then start the instant message tree to circulate the notice. Over the last 4 years both these practices have been discarded as students felt the VLE absorbed the function and gave added benefits such as access to two-way communication with the lecturers.

3^rd^ year student C, survey (2010): “On the class email, stuff would get deleted before we all saw it. Now information will be posted and kept on e-OT.”

3^rd^ year student D, survey (2010) commented specifically on the instant message tree: “Everyone is able to see announcements from lecturers so no one is left out*.*”

The above comments imply that students who had been involved over a few years felt marginalised in the earlier systems. It is clear that the students perceive the VLE to be a more equitable communication platform that provides peer-to-peer and student-lecturer engagement.

#### Instant gratification (Core category 2: outcomes BL integration)

There are both positive and negative aspects to this theme with the build-up of habits and expectations of what content and information will be posted and when.

Time was a factor in terms of students having anywhere, any-time access to their course information. Two students reflected on the positives of instant access and immediate performance results:

4^th^ year student C, survey (2012): “It is great that we have instant access to lecture notes, fieldwork information, marks etc.”

4^th^ year student D, survey (2012): “Quizzes and tasks on e-OT add novelty, plus instant results are a great benefit.”

Time was also expressed as a frustration when knowing that information is available but not immediately accessible because of poor bandwidth or power outages (see also Hurdles to BL in Lens 3).

3^rd^ year student E, survey (2010): “It is frustrating being unable to access when necessary due to internet difficulties”

Students noted that at times they anticipated that information would be posted within a (self-determined) time period and then finding that lecturers posted it later than expected. Many students agreed that test and assignment marks should be regularly updated so that they are able to monitor their own progress throughout the year. They suggested that lecturers post the notes before the lectures so that they can arrive prepared. It seemed that it was difficult for students to predict what information would be posted, and the students experienced some anxiety due to a sense of needing it all immediately. This issue was communicated to the lecturers during a curriculum meeting with the suggestion that they are alerted to the possible anxiety created and that communicating timelines would alleviate some of this tension.

### Critically reflective lens 3: our colleagues’ experiences (*the lecturer’s lens*)

The lecturers of the DOT were primary stakeholders in the PAR process and were actively involved in decision making, implementing actions and researching the outcomes. The lecturers were all female occupational therapists and ranged in academic experience from novice lecturer through to over 35 years.

#### Buy-in and goodness of fit (Core category 1: Drivers of change)

Collaborative decision making is a strong tool for creating buy-in to a concept and for driving the participation of all stakeholders. The strategic planning meeting in 2009 provided the platform for the lecturers of the DOT to agree that e-Learning was a good strategy to improve throughput, cope with increased student enrolment numbers, and create time for research. This set the platform for shared goals and beliefs which are a cornerstone of PAR [13, 14]. A sense of team cohesion was evident in the lecturers.

Lecturer D, individual interview (2013): “I think that your research took place when we had a good team in the department but I think having something like your project to keep things going and keep it exciting and have new things to do all the time has also been very nice for the team and given them a bit of a focus and a benefit to work towards something together. This kind of project may not work in a dissenting department.”

#### Support (Core category 1: Drivers of change)

The e-Learning team was physically located in the DOT. During interviews with the lecturers it frequently emerged that there was a sense of security in knowing that support from the project team was always available, whether to help set up a computer for media rich practical examinations or to help with showing how to do something on the VLE. This sense of availability and help at hand was a cornerstone to overcoming any hesitance on the part of the lecturers and consolidated the buy-in.

#### Just do it (Core category 1: Drivers of change)

In the early stages of the study, many lecturers simply used the VLE as a document repository and communication portal, but as some lecturers tried new learning activities, others realised that if they ‘just do it’, they started seeing new opportunities to be innovative in their teaching.

Ensuant lecturer B, focus group (2015): “In my module evaluation a student wrote “*Podcasts are the way of the future*” – They just loved that I did something different!”

Ensuant lecturer C, email February (2014): “Thought you’d want to do a happy dance with me too… (just thought I’d share how I’m using e-OT more…☺) I created my own lesson plan with multi-level jumps, videos & quizzes – have a look when you have time and let me know how I can improve it for next year!!”

These innovative lecturers showed that it can be done, which created a natural competitiveness that prompted other lecturers to revise their modules and start using new tools or ask the e-Learning team for assistance in doing something new.

#### Success (Core category 2: outcomes BL integration)

The determinant of success is usually considered to be an improvement in student performance (or at least outcomes that are equal to previous ones). Success has many forms; in this instance, it is seeing students actively engage with content that is sometimes tedious.

Lecturer B, email discussion (August 2015): “Firstly, although I find understanding the legislative background of employing people with disabilities interesting, it is REALLY boring to the students. […] So I thought I would take a chance using podcasts and the lesson plan format on e-OT to help me “flip the classroom” a bit. I turned the lecture period into an e-Learning time with a face-to-face feedback session thereafter. Knowing students and the boring nature of the content I did not have high hopes. Until I went outside to go to the cafeteria for a quick coffee and one of the students called me over to clarify something and I saw little groups of students sitting on the grass around their tablets listening to the podcasts. I promptly forgot about the coffee, as I was amazed at the level of engagement that I saw regarding this boring content. It seemed that the students were far more willing to engage and they actually understood the concepts far better than previously.”

Success can also be observed by improvement in the pass rate for the third-year paediatric assessment module, which we reported in Barnard-Ashton, Koch and Rothberg [28]. A cohort of 29 third year students (2010) who were novice to BL (introduced in that year) were compared to the 2011 third year students (*n* = 40) who were more experienced in integrating online activities with their PBL scenario exploration. Significantly higher (*p* ≤ 0.002) rate of accessing the VLE resulted in a marks shift effect size of d = 0.3 and the class average shifted from a fail in 2010 to a pass in 2011 [28].

Success for the lecturers is an increase in the research publication output of the department. In the decade prior to this study the average number of publications in accredited journals was 2.4 papers per year. In the current decade, the average per year is 6.7 papers. It is widely acknowledged that a number of factors have contributed to this success, namely the introduction of writing retreats, increased number of postgraduate students converting their research into published work and a special edition of the South African Journal of Occupational Therapy dedicated to the 70th anniversary of the DOT [29]. Many of the lecturers feel that the introduction of BL has improved their skills in the use of technology in the research and writing process, and that increased efficiency in the curriculum delivery through BL has afforded time take advantage of the other contributing factors.

#### Efficient use of resources (Core category 2: outcomes BL integration)

The lecturers concurred that one of the best aspects of the project has been the efficient use of time and conservation of paper.

Ensuant lecturer C, focus group (2015) “I do the whole train thing from Pretoria to here and that is a good solid hour that you are travelling, and with the iPad by the time I get to work all of my emails are answered, my to do list for the day done, so when I walk into the office I know exactly what it is I am supposed to be doing.”

Lecturer E, interview (2013): “To a degree it’s part of the culture that we need to have a paper version in a file somewhere, […] but in terms of meetings and all of those things I have gone more paperless […] I mean with an iPad I can file it somewhere […], the physical paper is irritating […], it has decreased my use of paper.”

Furthermore, lecturers felt able to provide students with feedback on their performance quicker, and good communication between lecturers and students meant fewer mishaps. Doing it electronically has a trail while paper in a pigeonhole can be claimed as lost.

#### Transparency of the curriculum (Core category 2: Outcomes BL integration)

A 4 year, full time degree has a complex interwoven curriculum that is difficult to fully conceptualise for both students and new lecturers. The VLE has become a central resource housing all the curriculum documents.

Ensuant lecturer D, focus group (2015): “When I started, that's how I learnt what the curriculum was. I spent hours on e-OT seeing how everything fits and connects and what the objectives were. It was such a fantastic tool.”

Lecturer E, focus group (2015): “I always say it is not a state secret what we want you to know - The students know what the standards are....it is there.”

#### Hurdles to bended learning (Core category 1: Drivers of change)

When looking at the drivers of change core category, consideration of the barriers of change emerged. Initially the lecturers’ biggest perceived hurdles to implementing BL were their own computing skills. Concerns over students being distracted by social media were also evident.

Lecturer A, focus group (2015): “I had one student watching a movie on his laptop, earphones in and all”

As students’ and lecturers’ computing skills and uptake of BL progressed, concerns shifted to infrastructure.

Ensuant lecturer C, focus group (2015): “For me the biggest challenge was the capacity of the system in terms of bandwidth and Wi-Fi access.”

Lecturer A, focus group (2015) commented on the power company’s scheduled power disruptions: “Load-shedding is creating a big problem but we are working around it.”

The lecturer’s attitudes to the use of BL have not been deterred by the perceived hurdles. Although infrastructure issues have disrupted online activities in the past, a campus-wide upgrade of the network and Wi-Fi is in process, while the upgrading of uninterrupted power supply to the network, the use of TCD’s with long battery life, and monitoring the load-shedding schedules have been cited as addressing the perceived barriers.

### Critically reflective lens 4: the theoretical literature

At the project inception the participants knew little of how to ensure the successful implementation of BL. Review of the literature pertaining to BL and PBL was crucial and the PM attended international congresses to gain insights into best practice. Presented here are the key innovation trigger articles and academic events.

#### Being evidence based - Learning theory (Core category 1: Drivers of change)

Siemens [30] recognised that the more learners gained access to the internet the greater their access to information. This has fundamentally shifted the way learners learn, as knowledge no longer has to reside within the person but can reside externally. He felt that traditional learning theories such as behaviourism, cognitivism and constructivism all focused the need for learning to be internal, and thus review of our learning theories was required. Using the constructs of chaos theory, networked learning, complexity theory and self-organisation, he developed the theory of Connectivism, which he coined as a “learning theory for the digital era”.

Siemens [31] defines Connectivism: “Connectivism is focused on the process of forming and creating meaningful networks that may include technology-mediated learning, [and] acknowledges learning that occurs when we dialogue with others […]. Connectivism is strongly focused on the linking to knowledge sources … not simply trying to explain how knowledge is formed in our own heads.”

Mentis (2008) supported the notion of Connectivism as the new age pedagogy for e-learning and BL environments. She considered the shift from traditional to emergent technology and the change in learning context from formal to informal as the platform for change in pedagogy towards self-directed learning which is ipsative and networked in nature [32]. In the last decade the support for Connectivism has grown and the impact of connected learning on teaching practice has emerged as a trend [33–36]. The theory of Connectivism thus formed the basis of decision making during the integration of e-Learning into the curriculum.

#### Being evidence based - international expert network (Core category 1: Drivers of change)

The PM attended two international congresses that profoundly contributed to the basis of knowledge development and expert network regarding BL. The EduLearn10 [37] congress in Barcelona exposed the eLearning Project team to international trends in BL and introduced the PM to Stephen Harris an Australian pioneer in BL [38]. This new network afforded the opportunity to visit The Sydney Centre for Innovative Learning (SCIL) and attend the Distance Education Hub (DeHUB) and Open and Distance Learning Association of Australia (ODLAA) Summit 2011–2021 [39]. This Summit exposed the PM to leaders in the field such as Terry Anderson and Grainne Conole. The visit to SCIL was a game changer – observing blended classes with adapted learning environments (camp fire, watering hole and cave spaces) at the intersection between pedagogy, virtual space and physical space [38]. Following their social media profiles and publications has given ongoing insight into their perspectives, research, presentations and innovations in learning, providing the PM with inspiration and ideas of BL integration opportunities for the study.

## Discussion

The results of the grounded theory analysis of the qualitative data collected during the 6 year process of medium impact [11] integration of BL at the program level [8] of the undergraduate occupational therapy curriculum, were presented using the Brookfield [16] critical reflection lenses to consider the perspectives of the stakeholders in the study. The results indicated two core categories to contribute to furthering our understanding of the process of integrating BL into an existing PBL curriculum.

### Core category 1: Drivers of change

Within the core category of “drivers of change” consideration should be given to the driving force and the antonymous perspective as a possible barrier to change when considering integrating BL into a PBL curriculum. A primary driver of change in this study was that of buy-in and goodness of fit. From both the lens of the PM and the lecturers, the sense of team cohesion, collaborative decision making, and the PM’s history with the DOT as a lecturer with perceived IT skills created a level of trust allowing full buy-in of the DOT to the process. An overall attitude of all stakeholders to “just do it” created a level of momentum within the process. Lecturers noted that the resilience to the naysayers and enthusiasm of the PM sparked them into trying new learning activities, which in turn created examples to others that if you just do it, it can be done. The PM lead by example in not waiting for an institutional VLE installation but finding an opportunity to collaborate with the Faculty IT for a local VLE.

Being evidence based through the lens of the literature provided the PM with the theoretical framework of connectivism and the expert role models to shape her strategies in guiding the process. While true connectivism is still what we strive for, evidence of students and lecturers accessing international experts (Table 1), collaborating via self-mediated online spaces (Lens 2) and the complete shift in how students access information indicates that connectivism is becoming embedded in the curriculum. Viewing the students as being primed for blended learning gives validity to this as a learning strategy, but for the lecturers and the PM a key driver is having support: management endorsing the project, budget to increase staff and technology capacity, technical support from Faculty IT and the e-Learning team were viewed as accessible and always willing to help lecturers and students. The primary hurdle to BL that remains within this context is the infrastructure as South Africa continues to be plagued with power outages [1], but with the rollout of the University network upgrade, higher capacity power backup are being installed and the use of mobile technologies is seen as a way forward.

In this study, we were fortunate that these drivers of change were possible and taking advantage of them has created a momentum and level of sustainability to the project. It would be valuable for others considering a similar process to evaluate their context for plausibility of creating these drivers of change.

### Core category 2: outcomes BL integration

The integration of BL was initiated through a strategic plan of the DOT to pro-actively combat the massification trends in South African higher education [1] and the threat this creates to workload and the ability of the DOT to produce research output. Over the 6 years of the project the lecturers and students have noted improved communication, curriculum transparency and efficient use of time and paper resources, which in turn accommodates the student need for instant gratification as suggested by Johnston [1]. Managing the expectation of instant access that BL created, reinforced the need for clear communication between the lecturers and students. Students felt reassured that they had the correct information and any-time access to all communication resulting in the use of BL becoming part of their study habits. The lecturers perceived success in seeing students actively engaged with BL activities and evidence that BL was contributing to improved pass rates [28]. The DOT has increased the average number of research papers produced per year when compared to previous years, and while this cannot be solely ascribed to the introduction of BL, it also cannot be excluded as a contributing factor. The integration of BL into the undergraduate occupational therapy curriculum has been valued as a game changer, receiving recognition within the University and local health education community.

### Critical reflection using the Brookfield [16] Lenses

Most models of critical reflection in practice focus on two main aspects: reflection-in-action and reflection-on-action [40], or provide categories of topics on which to reflect [15]. In health education critical reflection is largely focused on the development of critical clinical reflection in students as a skill [15]. Interestingly, Snowball and Mostert [41] presented their study on introducing blended learning in a first year accountancy course from three perspectives: the students, the lecturers and the educational technologist; inadvertently validating the stakeholder lenses included in the Brookfield [16] model. The reflection phases of using a PAR design should represent all stakeholder experiences [12, 13], the Brookfield [16] model synchronised well with the stakeholder groups and allowed for the influence of existing theory. This model entices a person to step into the embodiment of other stakeholder groups and set preconceptions aside, while acknowledging your own beliefs and positionality within the process. It is a useful tool in guiding reflection specifically in the context of changing practice in higher education.

## Conclusion

Reflecting, using the Brookfield [21] lenses, on the process of integrating e-Learning into an undergraduate curriculum considered the perspectives of the PM as a learner, the students’ experiences, the lecturer’s experiences and the contribution of BL theory. This process highlighted the key drivers of change and the outcomes evident of the contribution of BL to the stakeholders, leaving a strong sense of “there is no going back” among the lecturers and students of the DOT.

## Abbreviations

BL: Blended learning
DOT: Department of occupational therapy
IT: Information technology
PAR: Participatory action research
PBL: Problem based learning
PM: Project manager
TCD: Tablet computing device

## Organisation abbreviations

DeHUB: Distance Education Hub
FAIMER: Foundation for Advancement of International Medical Education and Research
ODLAA: Open and Distance Learning Association of Australia
SAFRI: Sub-Saharan Africa - FAIMER Regional Institute
SCIL: The Sydney Centre for Innovative Learning
